# Computational modeling of Adelta-fiber-mediated nociceptive detection of electrocutaneous stimulation

**DOI:** 10.1007/s00422-015-0656-4

**Published:** 2015-07-31

**Authors:** Huan Yang, Hil G. E. Meijer, Robert J. Doll, Jan R. Buitenweg, Stephan A. van Gils

**Affiliations:** Applied Analysis, MIRA Institute for Technical Medicine and Biomedical Technology, University of Twente, P.O. Box 217, 7500 AE Enschede, The Netherlands; Biomedical Signals and Systems, MIRA Institute for Technical Medicine and Biomedical Technology, University of Twente, P.O. Box 217, 7500 AE Enschede, The Netherlands

**Keywords:** Nociceptive pathway, Stimulus detection, Detection threshold, Stimulus parameters, Computational models, 92C50, 91E30

## Abstract

Sensitization is an example of malfunctioning of the nociceptive pathway in either the peripheral or central nervous system. Using quantitative sensory testing, one can only infer sensitization, but not determine the defective subsystem. The states of the subsystems may be characterized using computational modeling together with experimental data. Here, we develop a neurophysiologically plausible model replicating experimental observations from a psychophysical human subject study. We study the effects of single temporal stimulus parameters on detection thresholds corresponding to a 0.5 detection probability. To model peripheral activation and central processing, we adapt a stochastic drift-diffusion model and a probabilistic hazard model to our experimental setting without reaction times. We retain six lumped parameters in both models characterizing peripheral and central mechanisms. Both models have similar psychophysical functions, but the hazard model is computationally more efficient. The model-based effects of temporal stimulus parameters on detection thresholds are consistent with those from human subject data.

## Introduction

Increased insight into neurophysiological mechanisms of the nociceptive pathway may contribute to more reliable monitoring of chronification of pain and patient-tailored pain therapies (Dworkin et al. 2003; Baron 2006). To achieve this goal, a computational model of stimulus processing may be an in-dispensable tool. For instance, the model could provide a mechanism-based interpretation of experimental observations. In turn, this may explain or predict effects of pharmaceutical interventions in the nociceptive system. Another, prospective, use may be to estimate model parameters from measurements. The estimate might inform about the state of the nociceptive system and possibly indicate its malfunctioning, e.g., due to central sensitization, which could result in chronic pain (Latremoliere and Woolf 2009).

Hyperalgesia is a clinically important example of malfunctioning of the nociceptive system and is characterized as an increased response to a painful stimulus. It indirectly indicates central sensitization resulting from increased responsiveness, a decreased threshold, or changes in the receptive field (Sandkühler 2009; Latremoliere and Woolf 2009; Treede 2012). Quantitative sensory testing (QST) (Rolke et al. 2006) and electrical QST (Vaneker et al. 2005) may be used to demonstrate hyperalgesia by longitudinal measurements of thresholds. To study the underlying nociceptive system, one may use low-intensity electrocutaneous stimulation with intra-epidermal needle electrodes, since it was shown to recruit nociceptive A$\delta$-fibers preferentially, while bypassing mechanoreceptors (Inui et al. 2002; Mouraux et al. 2010; Steenbergen et al. 2012). Because of the low amplitudes, thresholds can only be determined from a sensory detection task rather than from a pain detection task.

Currently, there are few computational models of the nociceptive system (Britton and Skevington 1989; Britton et al. 1996; Xu et al. 2008; Farajidavar et al. 2008), but these focus on different stimulus modalities, i.e., thermal and tactile, and have a different outcome, i.e., pain sensation. As they do not include any stochastic component, they cannot simulate trial-to-trial variability. Hence, there is no neurophysiologically plausible model for a detection task with electrocutaneous stimuli. Detection tasks yield binary responses (yes/no). In general, this involves a two-alternative forced choice task which can be modeled with a drift-diffusion model (DDM) that accumulates noisy sensory evidence until a decision threshold is reached (Ratcliff and Rouder 1998; Bogacz et al. 2006; Ratcliff and McKoon 2008). The DDM may be interpreted as a stochastically spiking neuron model with a spike corresponding to the detection of the stimulus. Here we consider a detection task with electrocutaneous stimulation (Doll et al. 2014), where subjects only report the detected stimuli. The DDM also yields reaction times, but, as they are not recorded in the experiment, this is less relevant.

The electrical stimulus is a square-wave pulse train characterized by four parameters, i.e., the amplitude (*A*) and three temporal stimulus parameters: the pulse width (PW), the number of pulses (NoP) and the inter-pulse interval (IPI), see also Fig. 2. The detection threshold is the amplitude at which half of the stimuli are perceived (Treutwein 1995). This threshold was shown to depend on temporal stimulus parameters for various related stimulus modalities. The strength-duration curve describes the relationship between the stimulus amplitude and its pulse width to activate a neuron (Lapicque 1907; Mogyoros et al. 1996; Irnich 2010). As NoP increases, the threshold for first sensation of vibrotactile stimuli decreases (Nunziata et al. 1989). Gescheider et al. (1999) found that the decrease in the detection threshold of vibrotactile stimuli when decreasing IPI was due to superposition of neural responses. Other studies suggest that with multiple pulses, the afferent input to secondary neurons is increased by temporal summation (van der Heide et al. 2009; Mouraux et al. 2014). However, this effect should wear off for large IPI, and then, the subject may perceive both pulses independently (Zwislocki 1960; Viemeister and Wakefield 1991). This still increases the probability of perception. Hence, for a stimulus consisting of two pulses, a lower detection threshold is expected. However, the presence of temporal summation in the sensory detection task using nociceptive electrocutaneous stimuli has not been studied varying each single temporal parameter.

The aim of this study is to develop a computational model representing the essential peripheral and central mechanisms of processing of electrocutaneous stimuli. We want to replicate the experimental effects of all temporal parameters on detection thresholds within this model. To facilitate parameter estimation, the model should be computationally efficient and have as few parameters as possible. We take the drift-diffusion model as a starting point for trial-to-trial variability in psychophysical experiments. Although widely applied, a disadvantage of this model is that it is analytically intractable, especially for time-dependent input. The alternative is to use simulations, which is time-consuming. We follow an approach by Plesser and Gerstner (2000) to replace the stochastic problem by a probabilistic hazard model through an escape process. This leads to an efficient model for a detection task without reaction times.

As a motivation for the modeling, we first present preliminary experimental data from a human subject study. Next, we describe how electrical stimulation induces neural activity and leads to psychophysical responses. For the modeling, we incorporate peripheral fiber activation and sensory inputs at secondary neurons giving a drift-diffusion model. The activity can be close to threshold, and this is different from the original hazard model. We propose a different hazard function and show that our hazard model fits nicely to the drift-diffusion model with respect to the psychophysical functions. Next, we determine detection thresholds in the model and relate these to the experimentally observed thresholds. We discuss how the temporal parameters affect detection thresholds based on the model and conclude with further applications of the hazard model.

## Psychophysical human subject experiment

For illustrative purposes, we present data from a psychophysical human subject study with a yes–no detection task using electrocutaneous stimulation. The experiment considered in the present work is part of a more extended experiment. The psychophysical data and analysis in this manuscript illustrates the effects of temporal parameters on the detection task. A manuscript presenting the methodology and results of this human subject study in more detail is in preparation.

### Methods

Fifteen healthy human subjects participated in this study. The Medical Ethics Committee Twente approved all experimental procedures. All subjects provided written informed consent and were rewarded with a gift voucher after their participation in the experiment. Subjects visited the laboratory on two consecutive days. Experiments were conducted under the same conditions on each day. Electrical stimuli consisted of cathodic square-wave current pulses using an intra-epidermal needle electrode that was attached to the left forearm (Steenbergen et al. 2012; Doll et al. 2014). The electrical stimulus is characterized by the amplitude and three temporal parameters: the number of pulses, the interpulse interval and the pulse width. The experimental procedure lasted for ten minutes. Stimuli were selected according to an adaptive probing procedure (Doll et al. 2014). Subjects were instructed to press and hold a response button until a stimulus was detected. After the release, they were instructed to re-press the button after about a second. The inter-onset interval between two consecutively applied stimuli varied from 2 to 5 s. Stimuli with four combinations of temporal parameters, see Table 1, were presented in a pseudo-random order, but with an equal number of trials for each combination of temporal stimulus parameters. Logistic regression was used to obtain a detection threshold estimate from stimulus–response pairs per subject per day per combination of temporal parameters. A two-way repeated measures ANOVA was used to study the effect of parameter combination and the effect of study day on the detection threshold. Mauchly’s test was used to check violations of the sphericity assumption. Post hoc comparisons were performed without correcting for possible type I errors, as the analysis here is only meant to demonstrate the experimental phenomena.

**Table 1:** Four combinations of temporal stimulus parameters for the electrocutaneous pulse train stimulus

Index	1	2	3	4
NoP ($\#$)	1	1	2	2
IPI (ms)	-	-	10	50
PW (ms)	0.42	0.84	0.42	0.42

If $\text {NoP}=1$, then IPI is undefined

### Results

**Fig. 1:**
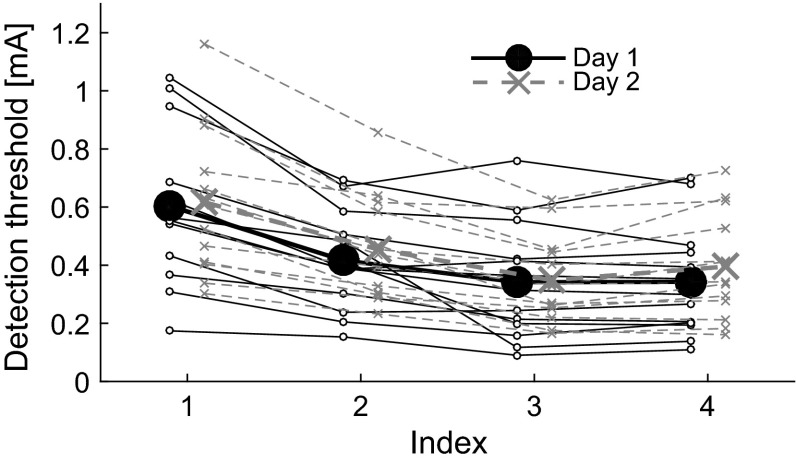
Detection thresholds for each subject, study day, and combination of temporal stimulus parameters (Table 1). The larger *solid circles* and *crosses* present the mean detection thresholds for day 1 and day 2, respectively

Two subjects were removed from the dataset due to technical issues on the second study day. The detection thresholds from individual subjects and the group are shown in Fig. 1. Mauchly’s test indicated that the assumption of sphericity for parameter combination had been violated ($\chi ^2(5) = .350; p = .047$). Therefore, the degrees of freedom were corrected as using the Greenhouse–Geisser estimates of sphericity ($\epsilon =.614$). The results show that there was a significant effect for parameter combination [$F(1.84, 22.10) = 66.82; p < .001$]. Study day had no significant effect on the detection thresholds [$F(1, 12) = .19; p = .67$]. Post hoc comparisons showed that increasing the pulse width (i.e., comparison between combinations 1 and 2) or the number of pulses (i.e., comparisons between combinations 1 and 3, 1 and 4, 2 and 3, and between 2 and 4) significantly reduced the threshold ($p \le .001$). The difference in threshold between the two two-pulse combinations 3 and 4 was at the significance level ($p = .051$).

## Computational modeling

**Fig. 2:**
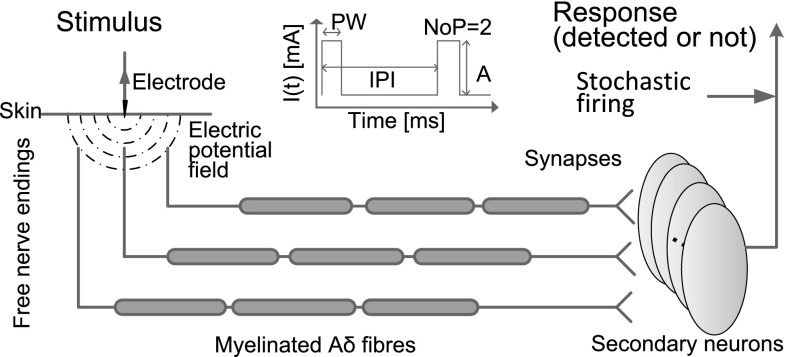
An electrode is attached to the skin of a subject to deliver pulse train stimulation. The *dot-dashed* concentric half *circles* represent the electric potential. Charging the nerve endings leads to traveling action potentials in the A$\delta$-fiber. The arrival of spikes at the presynaptic terminal triggers the release of the glutamate from the synapse resulting in an EPSC. The secondary neuron is charged, and the activity will converge upto the supraspinal part and lead to a binary response. Note that the number of signal channels is the number of secondary neurons, i.e., four in this diagram

Application of electrocutaneous stimulation charges nerve endings of A$\delta$-fibers. Action potentials are generated given sufficient stimulation. When this neuronal activity reaches the synapses that project to neurons in the dorsal horn, this triggers the release of neurotransmitter from the presynaptic terminal, inducing an excitatory postsynaptic current (EPSC). Consequently, the membrane potential of postsynaptic neurons increases and ultimately an action potential is generated. Sufficient neuronal activity leads to a supraspinal response where a subject responds ‘yes.’ Otherwise, the subject did not detect the stimulus as the neuronal activity was not sufficiently high. To quantitatively describe this detection process, signal conduction from skin to supraspinal part is modeled. First, we formulate the dynamic process in a single signal channel. Each signal channel consists of nociceptors, a synapse and a secondary neuron. Second, for the trial-to-trial variability, we include small background noise as additional input for secondary neurons. We also propose a convenient alternative based on escape noise (Plesser and Gerstner 2000). Lastly, we derive lumped models for the ascending nociceptive pathway by simplifying the multiple signal channels. The organization of the neuronal system is sketched in Fig. 2 with multiple signal channels.

### Activation of afferent fibers

For simplicity, we assume that the skin is a homogeneous medium with conductivity $c_0$, and the needle electrode is an infinitesimal point source generating an electric potential $V_\mathrm{e}$. Hence, applying electrocutaneous stimulation with a constant current amplitude *A*, the electric potential is given by $V_\mathrm{e}(r)=\frac{A}{4 \pi c_0 r},$ where *r* is the distance from the needle electrode. This electric potential generates the induced input to the A$\delta$-fibers. Usually, the effective input is the second spatial derivative of the potential along a fiber (Rattay 1999). However, in our experimental setup, relatively low amplitudes are applied, similar to (Mouraux et al. 2010). As a result, only the afferent fibers near the skin are recruited. In addition, the afferent fibers terminate in this region and mostly with the nerve endings perpendicular to the skin. For these nerve endings, the effective input at distance *r* is given by the first spatial derivative of the potential $I_A(r,t):= \frac{1}{c_1}\dfrac{\partial V_\mathrm{e}}{ \partial r}=-\frac{I(t)}{4 \pi c_0 c_1 r^2},$ where $c_1$ describes the resistance of nerve endings per unit length. For simplicity, we denote $c:=c_0 c_1$.

We use a cathodic electrode, so that the generated current *I*(*t*) is always negative, and the induced input depolarizes the membrane of nerve endings. In the sequel, we will write *A* instead of |*A*|. For simplicity, we take the nerve ending as a point in the three-dimensional space. Next, we model the dynamics of the membrane potential of the ending $V_1$ as a leaky integrator

1 $$\begin{aligned} C_1 \dot{V_1}=-G_1 V_1+I_A(r,t), \quad V_1(r,0)=0, \end{aligned}$$

where $C_1$ is the electrical capacitance of the nerve ending, and $G_1$ is the electrical conductance of the nerve ending. If $V_{1}$ exceeds a threshold $V_\mathrm{th}$, the fiber spikes. Given a single-pulse stimulus with duration PW, the maximal potential of $V_1(r,t)$ is a function of the distance

2 $$\begin{aligned} V_m(r):= & {} \max _{t \in [0,T]}V_1(r,t)\nonumber \\= & {} \frac{G_1^{-1} A}{4 \pi c r^2}\left( 1-\exp \left( -\frac{{\text {PW}}}{C_1 G_1^{-1}} \right) \right) , \end{aligned}$$

where *T* is the interval of a single trial. As the distance increases, the induced input decreases. So, the threshold $V_\mathrm{th}$ results in a critical value for the distance: All endings with a distance larger than this critical value are not activated. This critical value $r_c$ is computed by solving the equality $V_m(r_c)=V_\mathrm{th}$:

3 $$\begin{aligned} r_c=\left( \frac{G_1^{-1} A}{4\pi c V_\mathrm{th}} \left( 1-\exp \left( -\frac{{\text {PW}}}{C_1 G_1^{-1}} \right) \right) \right) ^\frac{1}{2}. \end{aligned}$$

**Fig. 3:**
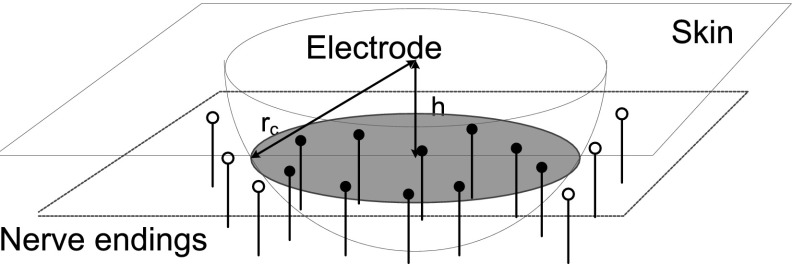
Illustration of the geometry of nerve endings under skin, a minimal depth is denoted by *h*. The endings with *solid tips* are recruited and those with *empty tips* are not recruited. The *gray surface* represents the recruited space, i.e., within critical distance $r_c$

So given the distance of a single nerve ending to the needle electrode, we can determine whether this ending generates a spike. Next, spikes from activated fibers drive the secondary neuron. We ignore the differences in the moments of action potential generation and also the arrival times of spikes at the secondary neuron. To describe the total input, we need to determine how many nerve endings are recruited. We assume that there is a homogeneous density $\rho$ of nerve endings under the stimulated tissue beneath the electrode and a lower bound on the depth *h* of the nerve endings from the skin, see Fig. 3. The number of the recruited endings $N_{r}$ is approximated to be proportional to the area of a circle within a sphere of radius $r_c$ at depth *h* and is given by

4 $$\begin{aligned} N_r=\pi \rho \left( r_c^2-h^2\right) H(r_c-h), \end{aligned}$$

where *H* is a Heaviside step function: $H(x)=1$, when $x\ge 0$; $H(x)=0$, when $x<0$. Here we approximate the actually integer number of recruited endings by a continuous quantity. If $N_r$ is small, this may be unsatisfactory. We discuss this later, but for a more elaborate modeling study on this issue, we refer to Mørch et al. (2011).

### Postsynaptic dynamics

We describe the postsynaptic potential (PSP) $V_2(t)$ of a secondary neuron also as a leaky integrator

5 $$\begin{aligned} C_2 \dot{V}_2= -G_2 V_2+I_p(t),\quad V_2(0)=0, \end{aligned}$$

where $C_2$ is the electrical capacitance of the secondary neuron, $G_2$ is the electrical conductance of the secondary neuron, and $I_p(t)$ is the EPSC. This EPSC is proportional to the potential gradient between the postsynaptic and AMPA reversal potentials, $(V_{2}-E_\mathrm{AMPA})$. As the inter-stimulus interval in repetitive electrocutaneous stimulation varied from 2 to 5 s, it is justified to assume that synaptic plasticity did not occur between trials. The IPI used for double-pulse stimuli is in the order of tens of milliseconds. This might involve short-term synaptic facilitation or depression at synapses from afferent fibers onto dorsal horn neurons. As recently reviewed in Luo et al. (2014), both may occur for various synapses, and the net effect is uncertain. Therefore, we do not include it here. Hence, we choose a simple reset-decay model for fast AMPA synapses (Roth and van Rossum 2009), whose impulse response is $g(t)= \bar{g} \exp \left( -t/\tau _s\right)$ for $t\ge 0$ with decay constant $\tau _s=1.5$ ms and maximal conductance $\bar{g}$ as a constant (Gabbiani et al. 1994). It is justified to set $V_{2}-E_\mathrm{AMPA}\approx V_R-E_\mathrm{AMPA}$ to some constant *K*, as we consider $V_2$ only below but close to the firing threshold $V_R$. The more the afferent fibers are activated, the more the presynaptic spikes are expected. To determine the precise timing of presynaptic spikes, both spike propagation and the variability of conduction velocity might play a role. First, the myelinated A$\delta$ fibers permit generated spikes recruited by relatively low stimulation frequency at nerve endings propagate along the nerves robustly. Second, the variability of conduction velocities of A$\delta$ fibers could lead to the variability in the arrival times at presynaptic terminals. However, the variability of conduction velocity for fibers from the same area is expected to be small. With a typical value of the conduction velocity for the A$\delta$ fiber 20 m/s and a distance of 50 cm, the spread of compound presynaptic spikes at the dorsal horn is expected to at most a few milliseconds. To determine postsynaptic activity, we do not take the variability of the conduction variability or the arriving times into account as the secondary neuron has a much larger time constant (Weng et al. 2006). These considerations encourage us to simplify presynaptic spikes from the activated afferent fibers by

6 $$\begin{aligned} u(t)=N_r \sum _{k=0}^{\mathrm{NoP}-1} \delta (t-k\, \mathrm{IPI}) \end{aligned}$$

with $\delta$ the Dirac delta function. Its convolution with $\mathrm{Kg}(t)$ gives the EPSC $I_p(t)$:

7 $$\begin{aligned} \begin{array}{rcl} I_p(t)&{}:=&{}(\mathrm{Kg}*u)(t)=\int _{0}^{\infty } \mathrm{Kg}(\tau ) u(t-\tau ) d\tau \\ &{}=&{}\frac{N_r\tau _s\bar{g}K }{\tau _s}\sum _{k=0}^{\mathrm{NoP}-1} \exp \left( -\frac{t-k\,\mathrm{IPI}}{\tau _s}\right) H(t-k\,\mathrm{IPI}). \end{array} \end{aligned}$$

Note that $N_r\tau _s\bar{g}K$ is a factor from afferent fibers, synapses and secondary neurons; the remaining $\tau _s$-normalized term facilitates the computation of $V_2$ by its convolution with the transfer function of the cascaded leaky integrator (5).

### Stimulus detection by randomly spiking secondary neurons

The activity evoked in afferent fibers induces postsynaptic activity in secondary neurons of the dorsal horn. This synaptic activity is noisy so that secondary neurons spike stochastically. We consider two descriptions of this random behavior: one stochastic and one probabilistic. We define that a stimulus is detected if at least one secondary neuron spikes.

#### Stochastic description: a drift-diffusion model

To describe the noisy dynamics of $V_{2}$, we employ the drift-diffusion model (Ratcliff and McKoon 2008). In contrast to the stimulation-induced presynaptic pulses, background presynaptic pulses are relatively weak. Assuming a large number of background pulses impinges on the neurons per membrane time constant, the net input to postsynaptic neurons can be modeled as additive white noise (Capocelli and Ricciardi 1971). Hence, the model (5) becomes a stochastic differential equation (SDE) with a deterministic term $I_p(t)$ and white noise input

8 $$\begin{aligned} C_{2} \mathrm{d}V_2= (-G_2V_{2}+I_p(t))\mathrm{d}t+\sigma _{\xi }\mathrm{d}W,\quad V_2(0)=0, \end{aligned}$$

where $\sigma _{\xi }$ is the noise strength and *W* is a standard Wiener process. We describe the binary outcome of ‘spiking or not’ of a single secondary neuron by

9 $$\begin{aligned} R_s:=H\left( \max _{t \in [0,T]} V_2(t) -V_R\right) , \end{aligned}$$

where we fix the trial interval $T=500$ ms. When $R_s=1$, it means that the neuron generated at least one spike within the trial interval *T*, otherwise none. We use the Euler–Maruyama scheme to obtain a single realization of the DDM with a fixed timestep of 0.01 ms (Kloeden and Pearson 1977). We approximate the probability of at least one spike $\varPsi _{D,s}$ by the average of $N=200$ realizations

10 $$\begin{aligned} \varPsi _{D,s}:=\Pr (\text {spike})=\overline{R_s}\approx \frac{1}{N}\sum _{i=1}^N R_{s,i}. \end{aligned}$$

At the level of the spinal cord, there are multiple secondary neurons that receive the stimulus-induced input. We assume this input is identical, but that the noise is independent. Then, for a population with *l* signal channels, the probability that at least one spike occurs is given by

11 $$\begin{aligned} \varPsi _D:=1-\left( 1-\varPsi _{D,s}\right) ^{l}. \end{aligned}$$

Note that this also defines the corresponding psychophysical curve for the DDM.

#### Probabilistic description: a hazard model

Escape noise (Plesser and Gerstner 2000) is another way to describe random spiking, given noise-free dynamics of secondary neuron (5) and (7). In other words, at each moment, the neuronal activity could exceed the firing threshold with a certain probability, even if the deterministic activity is below the firing threshold. We describe this stochastic firing with a nonhomogeneous Poisson process. For the time-varying firing rate of the Poisson process, we must choose a hazard function $\lambda _s$, which depends on the noise-free PSP. We take the widely used sigmoidal activation function for the hazard function

12 $$\begin{aligned} \lambda _s(t):=\lambda _s(V_2(t))=\frac{\lambda _h}{1+ \exp {\left( -{(V_2(t)-\alpha _h)}/{\sigma _h}\right) }}, \end{aligned}$$

where $\alpha _h$ is the activation threshold, $\lambda _h$ is the maximal firing rate and $\sigma _h$ is the slope parameter. Note that the activation threshold $\alpha _{h}$ has a different interpretation from the firing threshold $V_R$ in the DDM. In the DDM, given a realization of noise, the firing threshold determines the spiking in a deterministic way. In the hazard model, even if the noise-free PSP is below $\alpha _h$, there is still a probability to spike.

For a single neuron, the expected value of the number of spikes during this interval is given by

13 $$\begin{aligned} \lambda ^s_{T}:=\int _{0}^{T}\lambda _s(t)\mathrm{d}t. \end{aligned}$$

Thus, the probability of at least one spike in a single secondary neuron is given by

14 $$\begin{aligned} \varPsi _{H,s}:=1-{\mathrm {Pr}}\left( \text {no spike} |0\le t\le T\right) =1-\exp \left( -\lambda ^s_{T}\right) . \end{aligned}$$

For a population of neurons, similar to Eq. (11), we obtain the psychophysical function

15 $$\begin{aligned} \varPsi _H:=1-\left( 1-\varPsi _{H,s}\right) ^{l} = 1-\exp \left( -l\lambda ^s_{T}\right) . \end{aligned}$$

### Lumped models

We have built two models to represent the stimulus processing from electrocutaneous stimulation to random binary responses. However, these models have more than ten unknown physical quantities. To reduce the number of parameters, we introduce six lumped parameters for each model.

If we let the time constant of secondary neurons $\tau _2:= C_2 G_2^{-1}$, the lumped PSP $x:=G_{2}V_{2}/q$, the strength of white noise $\sigma :=\sigma _{\xi }/q$, the lumped EPSC $I_p^*:=I_p/q$ and the scaled firing threshold $\alpha _2:=G_{2}V_{R}/q$ where *q* is an arbitrary but nonzero constant, then the SDE can be rewritten as

16 $$\begin{aligned} \tau _2 \mathrm{d}{x}=\left( -x+I_p^*(t)\right) \mathrm{d}t+\sigma \mathrm{d}W, \quad x(0)=0. \end{aligned}$$

For a single neuron, the binary response is given by $R_s=H\left( \max _{t \in [0,T]} x(t) -\alpha _2\right)$, from which we can derive the psychophysical curves using Eqs. (10) and (11).

The gain factors in peripheral activation, central processing, and synaptic transmission are given by $\kappa :=\rho \left( 4 \pi c G_1 V_\mathrm{th} \right) ^{-1}$, *K* and $\bar{g} \tau _s$, respectively. All those gain factors are independent of the dynamics in underlying mechanisms. Hence, lumping those factors into the factor $q:=\bar{g} \tau _s \kappa K$, also see (7), we meet the requirement to get as few parameters as possible. Denoting the time constant of afferent fibers $\tau _1:=C_1 G_1^{-1}$, we can write $N_r=\kappa [f_A-\alpha _1]_+,$ where $[z]_+:=\pi zH(z)$ is a threshold-linear function, $\alpha _1:=4\pi c G_1 V_\mathrm{th} h^2$ is the lumped activation threshold, and $f_A:=A\left( 1-\exp \left( -\frac{{\text {PW}}}{\tau _1}\right) \right) =4 \pi c G_1 V_\mathrm{th} r_c^2$ is the amount of activation of afferent fibers.

Lumping the hazard model, we introduce $\alpha _{1},\tau _{1},\tau _{2}$ as for the DDM, the lumped activation threshold of secondary neurons $\alpha _L:=G_2\alpha _h/q$, the lumped slope parameter $\sigma _L:=G_2\sigma _h/q$ and the population firing rate $\lambda _L:=l\lambda _h$. It is now straightforward to compute the psychophysical function using the scaled noise-free dynamics $x^{0}(t):=G_2 V_2(t)/q$

17 $$\begin{aligned} x^{0}(t):= & {} \frac{\left[ f_A-\alpha _1\right] _+}{{\tau _{2}-\tau _s}} \sum _{k=0}^{\mathrm{NoP}-1}\left( \exp \left( -\frac{t-k\, \mathrm{IPI}}{\tau _{2}}\right) \right. \nonumber \\&\left. -\exp \left( -\frac{t-k\, \mathrm{IPI}}{\tau _s}\right) \right) H\left( t-k\, \mathrm{IPI}\right) \end{aligned}$$

by evaluating the integral

18 $$\begin{aligned} \varPsi _H=1-\exp \left( -\int _0^T \lambda (t) \mathrm{d}t\right) , \end{aligned}$$

where $$\lambda (t)=\lambda _L\left( 1+\exp \left( -\left( x^{0}(t)-\alpha _L\right) /\sigma _L\right) \right) ^{-1}$$.

To summarize, the lumped DDM involves six parameters: the threshold $\alpha _1$ and the time constant $\tau _1$ in the peripheral nervous system; the threshold $\alpha _2$, the noise strength $\sigma$, the time constant $\tau _2$ and the number of secondary neurons *l* in the more central system. Note that the lumped parameters $\alpha _2$ and $\sigma$ combine properties of the peripheral and the central system as they are scaled by *q*. For the lumped hazard model, we have the same $\alpha _{1},\tau _{1}$ and $\tau _{2}$, but the other three $\alpha _L$, $\sigma _L$ and $\lambda _L$ have a different interpretation. We will write ${{\theta }}_{D}:=(\alpha _1,\tau _1,\tau _2,\alpha _2,\sigma ,l)$ and ${{\theta }}_{H}:=(\alpha _1,\tau _1,\tau _2,\alpha _L,\sigma _L,\lambda _L)$ for the DDM and the HM, respectively.

### Comparison of the dynamics and psychophysical functions of DDM and HM

We formulated two models for the same detection task. These two models have the same fiber activation, but different formulations for spiking of secondary neurons. We present their dynamics and study how their psychophysical functions differ.

#### Activation of afferent fibers

**Fig. 4:**
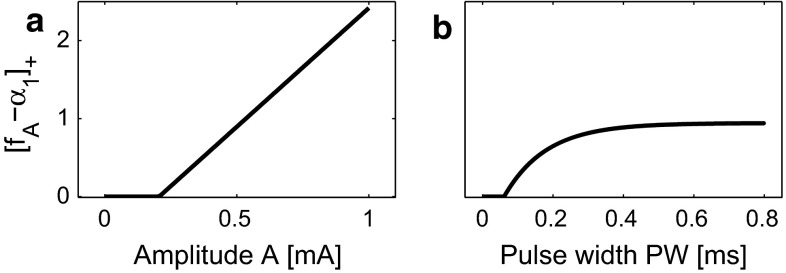
Activation of afferent fibers. **a** activation has a threshold nonlinear relation with amplitude *A*; **b** peripheral activity increases by increasing PW, eventually saturating

Fixing PW, the activation of afferent fibers $[f_A-\alpha _1]_+$ follows a threshold-linearity about *A*. Fixing the amplitude, the activation of afferent fibers grows by increasing PW saturating to rheobase. This is illustrated in Fig. 4 fixing parameter values $\alpha _1=0.2$ mA, $\tau _1=0.12$ ms and either (a) ${\text {PW}}=0.42$ ms or (b) $A=0.5$ mA.

#### Dynamics of secondary neurons

**Fig. 5:**
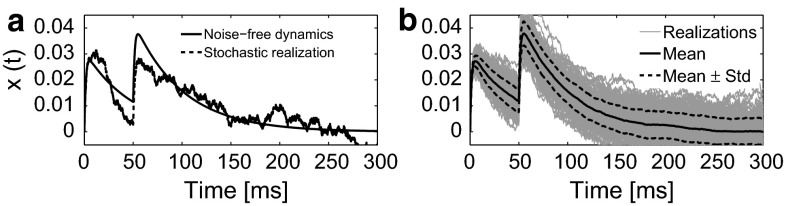
Stochastic dynamics of the DDM using a pulse train current input with ($A=1$ mA, $\text {NoP}=2$, $\text {IPI}=50$ ms, $\text {PW}=0.42$ ms). **a** Noise-free dynamics (*solid*) with $\sigma =0$ and a stochastic realization (*dashed*). **b** $N=200$ Realizations of the stochastic dynamics (*thin solid*). Statistics of the potential are also shown, mean (*solid*), and mean plus or minus the standard deviation (*dashed*). See text for system parameter values

We set stimulus parameters $A=1$ mA, $\text {NoP}=2$, $\text {IPI} =50$ ms and $\text {PW}=0.42$ ms and system parameters $\alpha _1=0.5$ mA, $\tau _1=0.1$ ms, $\tau _2=50$ mA, $\sigma =0.05$ A/s and $l=1$. The values of time constants $\tau _1$ and $\tau _2$ are based on (Mogyoros et al. 1996; Weng et al. 2006). In Fig. 5, we show realizations with and without noise.

**Fig. 6:**
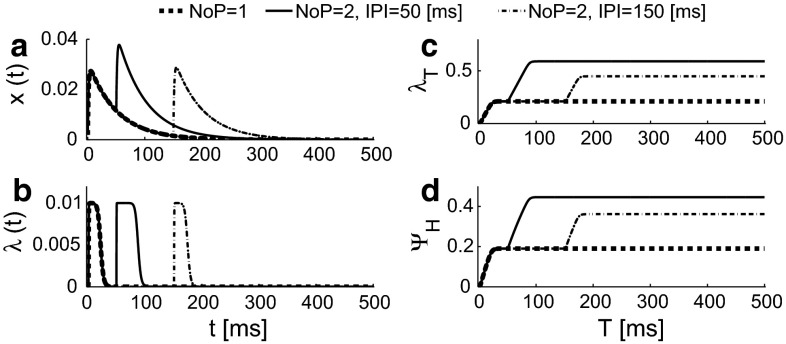
Activities of secondary neurons using three different stimuli with the same $\text {PW}=0.42$ ms: $\text {NoP}=1$ (*thick dashed*); $\text {NoP}=2$ and $\text {IPI}=50$ ms (*solid*); $\text {NoP}=2$ and ${\text {IPI}}=150$ ms (*dotted-dashed*). **a** Lumped PSP stimulated by an electrical train of two pulses with amplitude $A=1$ mA, ${\text {IPI}}=50$ ms and $\text {PW} = 0.42$ ms; **b** instantaneous firing rate; **c** the expected value of the number of spikes within a trial [0, *T*]; **d** psychophysical function value $\varPsi _H$ depending on *T*

To demonstrate the dynamics of the HM, we use the same parameter values but for the parameters associated with secondary neurons we use $\alpha _L=0.01$ A/s, $\sigma _L=0.001$ A/s and $\lambda _L=0.01$ kHz using three different stimuli with the same ${\text {PW}}=0.42$ ms: $NoP=1$ (thick dashed); $NoP=2$ and ${\text {IPI}}=50$ ms (solid); ${\text {NoP}}=2$ and ${\text {IPI}}=150$ ms (dot-dashed). The dynamics and the expected firing rate $\lambda _{T}$ are shown in Fig. 6. As the trial interval *T* is much larger than the time constant $\tau _2$, the psychophysical function value $\varPsi _H(A)$ does not change for larger values of *T*.

We implemented both models in MATLAB R2010b on a desktop with an Intel Core i7 processor. The time needed to evaluate a single psychophysical function value $\varPsi (A=0.1)$ was 0.21 s for the DDM using 4 cores and 0.0088 s for the HM. Hence, the HM is computationally much cheaper than the DDM.

#### Comparing psychophysical functions of DDM and HM

Since the psychophysical function of the HM is smooth, we start by choosing parameters that lead to experimentally plausible psychophysical functions for the DDM. Next, we fit the psychophysical function of the HM to the DDM at discrete stimulus amplitudes. The parameters $\tau _1, \tau _2$ and $\alpha _1$ are the same for both models, and hence, we will use the same values for the DDM and the HM. We do this for several combinations of the temporal parameters, see Table 2.

**Table 2:** Combinations of the temporal stimulus parameters

Index	A	B	C	D	E	F	G	H
NoP $(\#)$	1	1	1	2	2	2	2	2
IPI (ms)	-	-	-	10	20	50	100	150
PW (ms)	0.21	0.42	0.84	0.42	0.42	0.42	0.42	0.42

We use the relative fitting error to assess the difference between the HM and the DDM

19 $$\begin{aligned} E=\sum _{j}\frac{\sum _{i} \left( \varPsi _{D,j}(A_i)-\varPsi _{H,j}(A_i)\right) ^2}{\sum _i \varPsi _{D,j}(A_i)^2}, \end{aligned}$$

where *i* is the index of amplitudes, $A_i$ ranges from 0 to 2 with a step 0.01 mA, *j* is the index of the combination of temporal parameters, $\varPsi _{D}$ means the psychophysical function based on the DDM, and $\varPsi _{H}$ is the psychophysical function based on the HM.

**Fig. 7:**
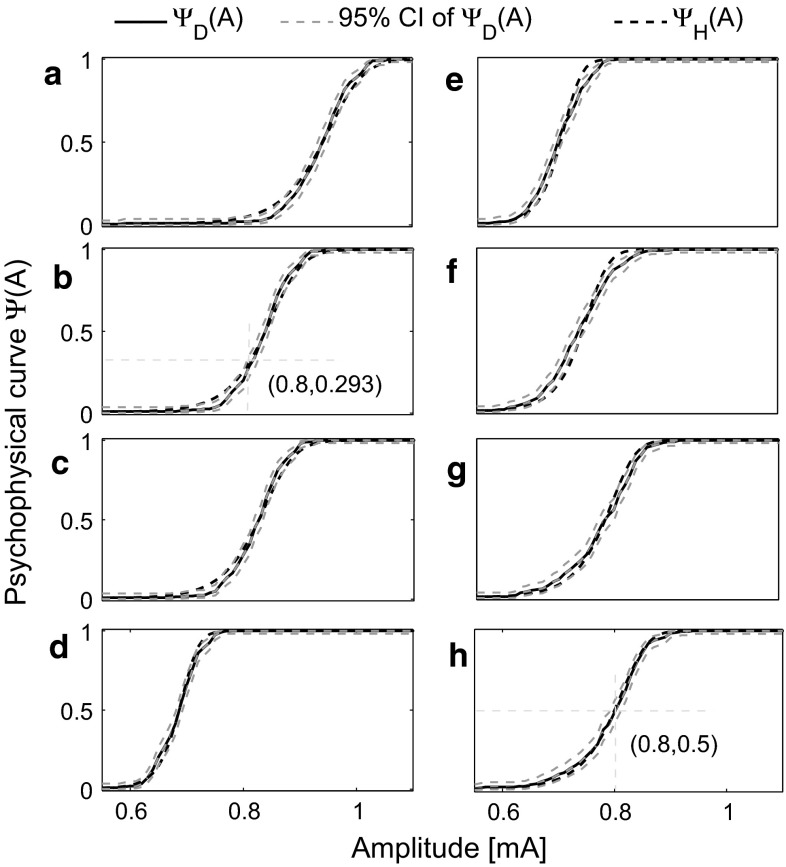
Using 8 different combinations of temporal stimulus parameters. Temporal parameters used in panels (**a**–**h**) correspond to combinations A–H in Table 2, psychophysical function values $\varPsi _D(A)$ using the DDM (*solid lines*) and best fitted $\varPsi _H$ by the HM (*dashed lines*) with fitting error $E=0.0029$. The common parameters are set to $\alpha _1=0.5$ mA, $\tau _1=0.1$ ms, $\tau _2=50$ ms, and $\tau _s=1.5$ ms. The parameters corresponding to neuronal variability are different in these two models. In the diffusion model, we set the values of parameter as $\alpha _2=0.02$ A/s, $\sigma =0.05$ A/s, and $l=1$, while the fitting results in $\alpha _L=0.0220$ A/s, $\sigma _L=0.0021$ A/s, and $\lambda _L= 0.4020$ kHz. The asymptotic behavior of the detection threshold with two independent pulses and its relation to the psychophysical curve with $\text {NoP}=1$ are illustrated by the thin dashed lines in panels (**b**) and (**h**)

For a particular choice of parameter values, the psychophysical functions after fitting are shown in Fig. 7. The realizations of the binary responses for exactly the same amplitude follow a binomial distribution. Hence, we compute the confidence interval (CI) using the Clopper–Pearson method (Clopper and Pearson 1934).

For stimulus combinations D and H, the psychophysical curves of the fitted HM lie within the 95 % CI of $\varPsi _D$. For other combinations, the fitted $\varPsi _H$ deviates negligibly from $\varPsi _D$, in particular for amplitudes far below or above the detection threshold.

We also study the fitting performance over a larger range of parameters for the DDM. We set two restrictions on the choice of the parameter values. First, we set ranges for the lumped threshold parameters so that the model detection thresholds are in the range of experimental observations, see Table. 3. For the time constants, the range of $\tau _1$ is set according to Mogyoros et al. (1996); the range of $\tau _2$ is 5–200 ms based on time constants of wide dynamic range neurons in rat dorsal horn (Weng et al. 2006). Second, as the electrode only delivers stimulation with low intensity, when $A=0$, the detection probability should be relatively low, i.e., near 0; when $A=2$ mA (the highest amplitude experimentally used), this probability should be close to 1. Therefore, our second restriction is $\varPsi _D(A=0)<0.35$ and $\varPsi _D(A=2.0)>0.65$.

**Table 3:** Parameter space for the DDM

Parameter	Lower bound	Upper bound
$\alpha _1$	0.05	1.00
$\tau _1$	0.01	0.50
$\tau _2$	5	200
$\alpha _2$	0.01	0.30
$\sigma$	0.02	0.20
$l \in {\mathbb {Z}}^+$	1	20

The upper three denote that the parameters are the same for the DDM and the HM

**Fig. 8:**
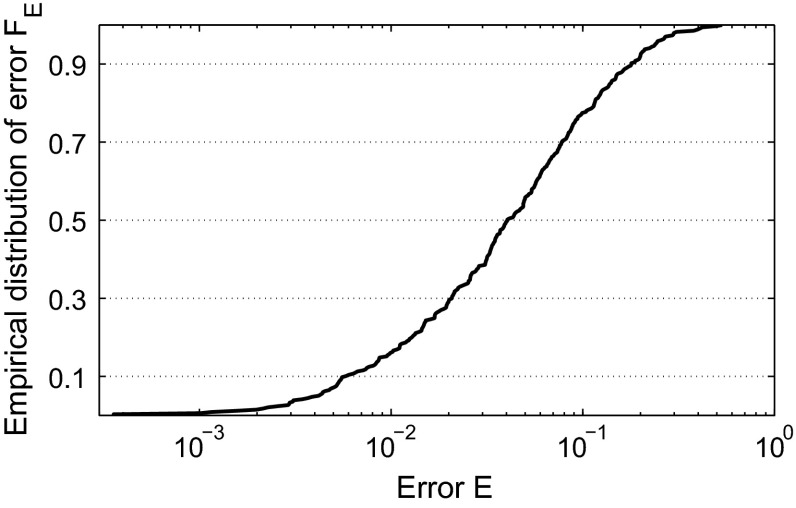
Distribution of the fitting error of the DDM by the hazard model with randomly chosen values of system parameters $\tau _1$, $\alpha _1$, $\tau _2$, $\alpha _2$, $\sigma$ and *l* in the DDM

With these restrictions, we apply a Monte Carlo method to study the fitting performance among the parameter space with the following steps. First, we sample a parameter vector ${{\theta }}_{D}$ within the parameter space randomly. Next, we verify whether the sampled parameter vector satisfies the second restriction; if yes, we continue, otherwise, we discard this sample and redo the first step to sample another parameter vector. Then, we compute $\varPsi _D$, estimate parameters $(\alpha _{L},\sigma _{L},\lambda _{L})$ for the HM and compute the fitting error *E*. We do these steps 500 times so that we obtain a set of errors. Finally, we determine the empirical distribution of the fitting error denoted by $F_E$, see Fig. 8.

This plot describes how well the HM can be fitted to the DDM in the parameter space. The ideal result of fitting would be $$E\equiv 0$$ while, in practice, model differences cause differences between psychophysical functions. The goodness of fit can be assessed by looking at the error level when $$ F_E$$ crossing 50 %, i.e., the level which half of fittings do not exceed. According to Fig. 8, we have 50 % to have a fitting error $$E\le 0.040$$. This result shows that psychophysical functions of the HM are similar to those generated by the DDM, for most choices of the parameters of the DDM.

## Effects of temporal stimulus parameters on detection thresholds

Detection thresholds are important psychophysical quantities and they depend on stimulus parameters. We compare model-based thresholds with experimental values. We give a neurophysiological interpretation of the effects of temporal stimulus parameters on detection thresholds using the two models.

We can determine the threshold $A_{50}$ in a model by solving $\varPsi (A_{50})=0.5$. This definition only makes sense if $\varPsi (A=0)<0.5$, i.e., spontaneous activation is unlikely in the absence of stimuli. Therefore, for the HM, we impose the condition $T\lambda _{L}<\ln (2)(1+\exp (\alpha _{L}/\sigma _{L}))$. If this is satisfied, it is straightforward to obtain the unique threshold as the psychophysical function is a monotone function of the stimulus amplitude in the hazard model. For the DDM, it is nontrivial to derive such a condition as it would require to evaluate infinite-dimensional integrals for which no closed-form formula exists. Hence, for the DDM, we rely on (many) simulations to find $\varPsi _{D}$ and determine $A_{50}$ by interpolation.

**Fig. 9:**
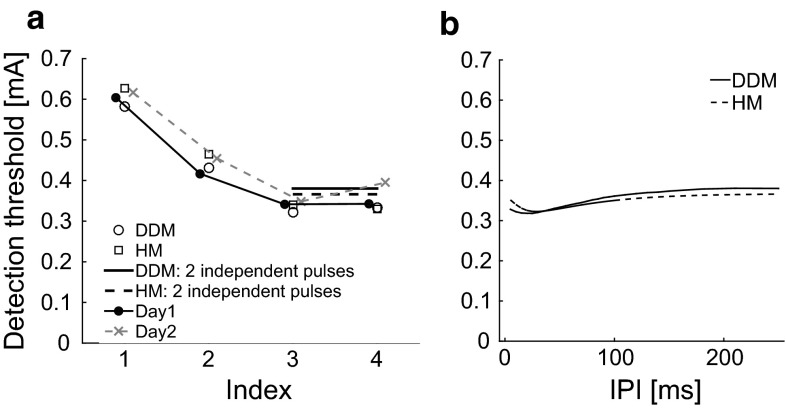
Dependence of detection thresholds on temporal parameters. **a** Experimental detection thresholds (*solid circles* and *crosses*) as in Fig. 1 and model-based thresholds (DDM: *open circles*, HM: *open squares*). The index refers to the combinations of Table 1 and see text for parameter values. The *horizontal lines* for ${\text {NoP}}=2$ indicate the asymptotic value $A_{2,50}$ for two independent pulses (DDM: *solid line*, HM: *dashed line*). **b** Non-monotone dependence of two-pulse threshold $A_{2,50}$ on IPI

We now consider the experimentally observed detection thresholds using our models. Given a parameter set and the experimental combinations, for which we refer to Table 1, we can compute the detection thresholds of the models. Varying parameters systematically, we found parameter sets ${{\theta }}_D=(0.06,0.4,50,0.031,0.09,8)$ and ${{\theta }}_H=(0.06,0.4,50,0.006,0.001,0.01)$ such that detection thresholds of both models were close to the experimental values, see Fig. 9a. This illustrates that both models replicate the experimental phenomena, i.e., increasing PW and *NoP* decreases detection thresholds. From Fig. 4, we see that increasing PW leads to more activated fibers. This increases the input to secondary neurons making them more likely to spike, hence decreasing the threshold. The models also explain why more pulses lower the detection threshold. For the HM, the activity $x^{0}$ after the first pulse returns to base-line if IPI is large. Hence, for two independent pulses, the expected firing rate doubles, i.e., $\lambda _{T}(A,\text {NoP}=2)= 2\lambda _{T}(A,\text {NoP}=1)$, illustrated in Fig. 6c. So we see $\varPsi _{H}(A,\text {NoP}=2)=1-(1-\varPsi _{H}(A,\text {NoP}=1))^2>\varPsi _{H}(A,\text {NoP}=1)$. The resulting $\varPsi _{H}(\text {NoP}=2)$ is shifted to the left and steeper. This reflects that it is more likely to detect at least one of the two independent stimuli. A similar reasoning holds for the DDM as a spike is more likely to occur, as two pulses increase the probability for the stochastic PSP to exceed the firing threshold. We can also use the latter relation to predict the threshold $A_{2,50}$ for two pulses with large IPI based on the psychophysical function of one pulse giving the equation $\varPsi _{H}(A_{2,50},\text {NoP}=1)=1-\frac{\sqrt{2}}{2}$. The detection thresholds $A_{2,50}$ computed from this relation is indicated in our comparison of DDM and HM in Fig. 7b, h and for the experimental data by the horizontal lines in Fig. 9a.

**Fig. 10:**
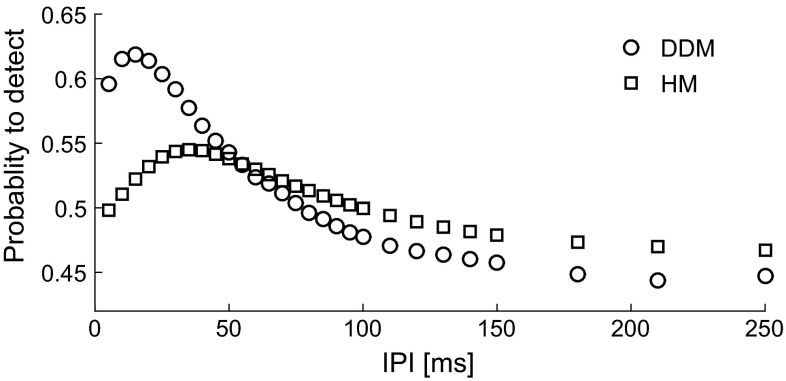
Model-simulated non-monotone effects of the IPI on the probability to detect when in the DDM (*circles*) and the HM (*squares*) for $\text {NoP}=2$. The amplitude is fixed as $A=0.35$ mA, and values of system parameters are identical to those used in Fig. 9

In addition, changing IPI from 10 to 50 ms, we see that both experimental and model-based thresholds do not vary much. We have computed the thresholds for varying IPI, see Fig. 9b. This relation exhibits a value of IPI with minimal $A_{50}$. At this value, temporal summation of the PSP maximizes the expected value of the number of spikes for the hazard model. Likewise, for the drift-diffusion model it increases the time window where the dynamics is just below the threshold. Therefore, also for the DDM we find a value of IPI that minimizes $A_{50}$. Now for the data, the experimentally used IPI in Table 1 may have been either on both sides of such an optimal value or on the long flat tail, see the horizontal lines in Fig. 9a. In both cases, the models explain why the effect of IPI on threshold may be nonsignificant and at best small. The psychometric function describes the probability to detect stimuli at various amplitudes. To demonstrate the effect of temporal summation, one can fix the amplitude and compute this probability as a function of IPI. In Fig. 10, we show similar non-monotone trends of the detection probability as IPI is increased for both models for the same parameter values. These simulated trends are in line with the IPI effect in an eyeblink response task (Blumenthal et al. 1987).

## Discussion

We modeled a detection task with an electrocutaneous pulse train stimulus. We derived a stochastic drift-diffusion model and a probabilistic hazard model with six lumped parameters characterizing the underlying neurophysiological mechanisms. Using the models, we explained the effects of temporal stimulus parameters on thresholds in a human subject study. Both models have similar psychophysical curves, but the hazard model is computationally more convenient and hence more suitable for follow-up studies.

### Effects of temporal parameters on detection thresholds

The pulse train stimulus has three temporal stimulus parameters. Increasing PW, more nerve endings are activated, see Fig. 4b, which increases the activity of secondary neurons. This lowers the detection threshold in accordance with many other studies on neural activation (Mogyoros et al. 1996; Irnich 2010). Increasing NoP, we noted two effects depending on the value of IPI. For large IPI, each pulse may be perceived independently, increasing the detection probability. This is known as probability summation (Zwislocki 1960; Gescheider et al. 1999). For shorter IPI, temporal summation of neural responses may further decrease the detection threshold. Our model differs from an earlier one by Zwislocki (1960), as we describe also trial-to-trial variability, accounting for a wide range of IPI values. Our experimental detection thresholds showed only a small increase when changing IPI from 10 to 50 ms, which was at the significance level. This differs from the phenomenon observed in another study of tactile sensory processing (Gescheider et al. 1999). One possible explanation is based on the non-monotonic relationship from our model simulations due to the following reasons. First, tactile sensory processing relies mostly on A$\beta$-fibers. The A$\delta$-fibers activated by the electrocutaneous stimulation differ in two aspects: their intrinsic neurophysiological properties and the neurophysiological characteristics of central neurons located in different laminae in the dorsal horn (Todd 2010). Such differences could be reflected by the time constant of 200 ms for neural response used in Gescheider et al. (1999), which is much larger than the time constants of afferent fibers and secondary neurons in the nociceptive system (Mogyoros et al. 1996; Weng et al. 2006). Second, Gescheider et al. (1999) utilized a merely deterministic model for the neural response, which did not account for the noisy neural activity with paired pulses. Third, the agreement on the non-monotone IPI effect on the detection probability between our model simulation and (Blumenthal et al. 1987) further supports our hypothetical explanation of the small IPI effect on detection thresholds. Future experiments could use a wider range for IPI to study the effect of IPI in more detail.

Other processes, such as threshold noise (Coombes et al. 2011), could also account for trial-to-trial variability. It is unclear what the plausible autocorrelation of the noise should be. Including threshold noise would also increase the number of lumped parameters by introducing parameters to characterize the autocorrelation. In addition, we encounter the same difficulty as for the DDM when we want to determine the distribution of the first passage times (FPTs) to compute the psychophysical function. Because of a lack of an analytically tractable expression of the distribution of the FPTs (Ricciardi and Sato 1986; Di Nardo et al. 2000), model-based detection thresholds can only be simulated by generating a large set of realizations of threshold noise. Hence, a model considering threshold noise would be computationally expensive. This restricts the usage of a model with threshold noise in follow-up studies, e.g., parameter estimation.

### Interpretation of lumped parameters

In both models, six lumped parameters characterize peripheral and central mechanisms. In the hazard model, the time constant $$\tau _1$$ and the threshold $$\alpha _{1}$$ affect the activation of peripheral fibers. The time constant $$\tau _2$$ and the firing rate $$\lambda _L$$ describe central properties. The threshold $$\alpha _{L}$$ and the slope parameter $$\sigma _{L}$$ depend on peripheral and central components. The physical quantities *h*, $$C_1$$, $$C_2$$, $$\alpha _h$$, and $$\sigma _h$$ occur solely in the lumped parameters $$\alpha _1$$, $$\tau _1$$, $$\tau _2$$, $$\alpha _L$$, and $$\sigma _L$$, respectively. When one of these lumped parameters changes, one can attribute this to the corresponding physical quantity. For other physical quantities, this may not be the case. We discuss two pathological phenomena: hyperalgesia and central sensitization. When considering several possible causes of hyperalgesia (Sandkühler 2009) with either a change in excitability of afferent fibers or secondary neurons or a change in synaptic strength, we can determine the corresponding change in the lumped parameters. Membrane excitability of afferent fibers and secondary neurons, and synaptic strength are characterized by $$\kappa $$, *K* and $$\bar{g}$$, respectively. In peripheral activation, increased peripheral excitability $$\kappa $$ reflects in the simultaneous decrease in $$\alpha _{1} \propto \kappa ^{-1}$$, $$\alpha _L\propto \kappa ^{-1}$$ and $$\sigma _L\propto \kappa ^{-1}$$. In central processing, the product of $$\bar{g}$$ and *K* can be considered as the compound excitability of synapses and membranes of secondary neurons. As the lumped parameters $$\alpha _L,\sigma _L $$$$\propto (\bar{g}K)^{-1}$$, lower values of $$\alpha _L$$ and $$\sigma _L$$ indicate a higher compound excitability. However, individual contributions of $$\bar{g}$$ or *K* to the compound excitability cannot be distinguished from these lumped parameters. In addition, central sensitization can manifest itself as a reduced outward flux of potassium ions of secondary neurons (Latremoliere and Woolf 2009). This inhibition can be cast in a decrease of $$G_2$$. Such a decreased value of $$G_2$$ would result when $$\tau _2 \propto G_2^{-1}$$ increases, and both $$\alpha _L \propto G_2$$ and $$\sigma _L \propto G_2$$ decrease simultaneously. So different patterns of changes of these lumped parameters reflect distinguishable changes in either peripheral activation or central processing. Hence, these lumped parameters may be used in a patient-specific interpretation of (mal-)functioning in the nociceptive system. In addition, such understanding of effects of parameters would be used to guide new experimental design based on the model, such as predicting the effect of certain medication on thresholds based on modulation of synaptic efficiency in the dorsal horn. When such experimental measurements with detection thresholds under a perturbed nociceptive system become available, in turn, our understanding of parts of nociceptive processing might advance as well. As the model behavior depends nonlinearly on multiple parameters, effects of these system parameters on detection thresholds will be a subject of future work.

### Model identifiability

For a practical application of our modeling study, one needs to infer system parameters from psychophysical measurements, as most of the lumped parameters are not measurable in a direct way. Obviously, we will not be able to determine the physical quantities, but only the lumped parameters. Here we have chosen parameter sets to match model-based thresholds to experimentally obtained values, since an appropriate parameter estimation procedure is currently lacking. Standard system identification techniques are based on time-varying input and output (Ljung 1999). In contrast, QST for pain diagnosis and monitoring yields psychophysical characteristics like thresholds rather than time-varying measurements (Wilder-Smith and Arendt-Nielsen 2006; Doll et al. 2014). In future work, we will investigate whether one can use these characteristics or stimulus–response pairs for system identification. The hazard model provides a good starting point as it is efficient and captures the experimental effects of temporal stimulus parameters. However, different from the logistic curve which is a generalized linear model, the nonlinearity in the HM challenges the assessment of model identifiability. Prior to performing parameter estimation, the identifiability of the hazard model should be explored, i.e., a unique estimate can be determined provided sufficient information is available (Bellu et al. 2007; Raue et al. 2009). In our setting, it is a challenge to design suitable combinations of temporal stimulus parameters.

### Model extensions

So far, we have considered the essential neural mechanisms of stimulus processing in the ascending pathway. With respect to fiber activation, our model could be extended in two ways. First, we simplified the discrete number of activated nerve endings by the continuous variable $N_r$ (Eq. 4). However, as both the strength of the induced electric field and the density of nerve endings determine this number, it is a challenge to improve this approximation for human subjects. If the A$\delta$-nerve endings would be sparsely distributed, we recommend to minimize variations of the electrode–skin interface in experiments. Second, we assumed robust spike propagation and small variability in conduction velocity, because of the myelination of normal A$\delta$-fibers. However, a demyelinating disease could amplify the contribution of these two factors on the postsynaptic activity, making these two terms necessary. Hence, on the one hand, more work is required to adequately describe the mechanisms for patients with a demyelinating disease; on the other hand, using our model, we suggest to use the presence of demyelinating disease as an exclusion criterion. Short-term plasticity is another process relevant for sensory synaptic transmission. We have not included this mechanism in our current model for several reasons. First, experimental evidence is collected for various synapses between afferent fibers and different laminae in the dorsal horn, see the review (Luo et al. 2014). As both synaptic depression and facilitation may occur, the net effect is uncertain. Second, our model already explains the effect of the IPI on detection thresholds. Short-term plasticity may interfere with temporal summation, but both the data and the model suggest a small effect of the IPI. Nevertheless, if new experimental data provide conclusive evidence on the net effect due to the short-term plasticity, such an effect can be effectively modeled by modifying (7) such that $\bar{g}$ depends on IPI. Third, chronification of pain states is accompanied by the long-term plasticity, e.g., central sensitization. Such clinical relevance draws more attention on the long-term plasticity rather than short-term forms. To induce and maintain central sensitization, NMDA receptors (NMDAR) play an important role (Woolf and Thompson 1991). Our models do not represent signal transduction, like protein kinase in postsynaptic neurons induced by the influx of calcium ions via NMDAR (Latremoliere and Woolf 2009). Such mechanisms affect membrane excitability of secondary neurons and synaptic strength. Their compound effect is characterized by the two lumped parameters $\alpha _L$ and $\sigma _L$. Hence, parameters in the hazard model can still reflect the (mal-)functioning caused by involvement of NMDAR. An extended model representing more mechanisms due to NMDAR might increase insights into central sensitization. However, the substantially increased number of parameters for complex signaling pathways would challenge parameter estimation using psychophysical data from QST.

In addition, stimulus processing can be modulated by the descending pathway, but under normal circumstances it is inactive. Yet, it is clinically important as its malfunctioning may be related to chronic pain (Yarnitsky 2010). The descending pathway may be activated by a conditioning stimulus such as the cold pressor test (CPT) through conditioned pain modulation (CPM) (Pud et al. 2009; Yarnitsky et al. 2010). It has been shown that the CPT leads to temporally increased detection thresholds (Doll et al. 2014). It is possible to incorporate descending inhibition to stimulus processing along the ascending pathway although the precise form, multiplicative due to shunting or additive due to normal inhibition, is unknown. If parameters of the ascending system can be estimated, it would then encourage to identify the descending pathway.
